# Medical Department of the Cincinnati College

**Published:** 1837

**Authors:** J. B. Rogers

**Affiliations:** Dean


### Art. IX.—Medical Department of Cincinnati College.

The session commences the last Monday of October, and ends the last day of February.

*Special and Surgical Anatomy*, by Dr. M'Dowell.

*General and Pathological Anatomy and Physiology*, by Dr. Gross.

*Surgery*, by Dr. Parker.

*Obstetrics and the Diseases peculiar to Women and Children*, by Dr. Rives.

*Chemistry and Medical Jurisprudence*, by Dr. Rogers.

*Materia Medica and Pharmacy*, by Dr. Harrison.

*Theory and Practice of Medicine*, by Dr. Drake.

*Dissections and Practical Anatomy*, by Dr. Trimble.

*Clinical Instruction in the Cincinnati Hospital*, by Drs. Drake, Parker & Rives.

Professor Parker, now in Europe for the purchase of additional books and apparatus, will return in October.

Dr. Trimble will open the rooms for Practical Anatomy on the 1st of October, and Professor M'Dowell will at the same time commence a preliminary course of Osteology.

Expenses.—Tickets of the Professors, $15 each—Matriculation, $5—Library Ticket, (optional,) $3—Hospital Ticket, $5 —Anatomical Rooms, $10—total, $125. Respectable boarding and lodging can be had at $3 a week.

As we have no national circulating medium, the Faculty consider it proper to give notice, that they will receive from students, at par, the current bank bills of the different states in which they respectively reside:

By order of the Faculty,

J. B. ROGERS, Dean.

